# What drives career choice in allied health professions in England? Insights from a national questionnaire

**DOI:** 10.1186/s12913-025-13591-y

**Published:** 2025-12-25

**Authors:** Lucy Wallis, Maja Palmer, Rachel Locke, James Faulkner, Carrie Biddle, Beverley Harden

**Affiliations:** 1 Population Health Sciences, University of Bristol, Bristol, UK; 2 University of Southampton, Southampton, UK; 3 University of Winchester, Winchester, UK; 4 Primary Care Research Centre, University of Southampton, Southampton, UK; 5 NHS England Southwest Region, Plymouth, UK; 6 NHS England, Leeds, UK

**Keywords:** Allied health professionals, Students, Career choice, Recruitment, Questionnaire, Sources of influence

## Abstract

**Background:**

In July 2025, the NHS (National Health Service) Plan was published signalling the need for strong workforce supply pipelines to support shifts towards care in the community and early access to diagnostics and treatment. Allied Health Professionals (AHPs) are vital to diagnostic pathways, in and out patient care, rehabilitation and home care as well as public health and prevention, thereby keeping people independent, and participating in society. Conducting a national analysis of AHP career choices can inform targeted recruitment strategies for the AHP workforce and individual professions to meet this need. The aim of this study was to understand the influence of sources driving career choices and the stage in which AHP students in England choose their profession to identify recommendations for stimulating demand for AHP careers.

**Methods:**

An online questionnaire was disseminated to undergraduate and postgraduate students and apprentices on pre-registration AHP courses in England.

**Results:**

A total of 1,318 participants completed the questionnaire with all professions represented. The majority of AHP students make their career choice after a first career (32.5%) or during college/sixth form (26.6%). *Conducting your own research*, *impact of a role model* and *university open days and information* were the most influential sources. Conversely, a *careers advisor*, or *print and television adverts* appeared as some of the least influential sources.

**Conclusions:**

Qualified AHPs should continue to be utilised and better supported as role models in particular in acting as ambassadors when disseminating learning about their profession to patients, for schools and in outreach work to target career changers. The role of universities in acting as a source of influence is wide-ranging and includes engaging more with schools to promote the AHPs as part of the curriculum and engaging with current university students about an AHP career choice. Careers advisors should be supported to deliver effective career advice about the AHPs. These three stakeholders should be backed by the NHS, AHP professional bodies and healthcare organisations to act as key sources of influence.

**Supplementary Information:**

The online version contains supplementary material available at 10.1186/s12913-025-13591-y.

## Background

The World Health Organisation projects a shortfall of 10.2 million health workers by 2030 [1]. Allied Health Professionals (AHPs) are one of the largest groups of healthcare professions and their skills are central to building resilient healthcare. AHPs are professionally autonomous practitioners who assess, diagnose, treat, and discharge patients [2] with diverse and distinct professional skills and knowledge [3, 4]. The categorisation of AHPs varies between countries but the group tends to exclude medicine and nursing. Definitions may also differ within a country: for example, in Australia each territory has a different number of professions under the banner of Allied Health [5] and include professions such as social workers and psychologists. Within the state of Victoria there are 27 listed AHP professions, of which only some are patient-facing [5]. In Canada, the AHP workforce includes dental hygienists and chiropractors [6] and in the United States, medical technologists are recognised as AHPs whereas osteopaths and podiatrists are not [5]. In England, the definition aligns with the 14 registerable titles for AHPs which covers 15 specific roles recognized by NHS (National Health Service) England (the NHS is the public sector health service): art therapists, dietitians, dramatherapists, music therapists, occupational therapists (OT), operating department practitioners (ODP), orthoptists, osteopaths, paramedics, physiotherapists, podiatrists, prosthetists/orthotists, diagnostic and therapeutic radiographers, and speech and language therapists (SLT).

There is a need in England for a sustainable supply of future AHPs [7, 8]. There are approximately 221,125 AHPs according to current HCPC (Health and Care Professions Council) registrant figures [9], although this does not include the number of registered osteopaths with the General Osteopathic Council. The AHPs comprise the third largest clinical workforce in the NHS [8]. However, the size of each profession varies, with physiotherapists comprising the largest workforce and orthoptists and prosthetists/orthotists the smallest [10].

The 2023 NHS Long Term Workforce plan [7] outlines the long-term strategy in England to address current and future NHS workforce shortages. Stimulating interest in AHP careers will be essential for securing their future supply [8]. The need for effective recruitment to the AHPs is not limited to England, with a survey in the United States revealing that 85% of 1005 healthcare facilities were experiencing a shortage of AHPs [11]. Although progress has been made in growing the profile of AHPs [8, 10], this can still be developed and embedded. Health Education England (HEE) (now NHS England) highlighted the importance of raising awareness and understanding of who the AHPs are, both as individual professions and as a collective, to support recruitment into the professions [8]. A survey [12] with 5,259 young people aged 16–26, commissioned by Universities UK (United Kingdom) and analyzed by the Nuffield Trust, highlighted the critical need to raise awareness of AHPs and the breadth of healthcare careers available. The findings revealed that only 20% of respondents reported being ‘very familiar’ with NHS career options beyond roles as doctors or nurses, underscoring a significant gap in public knowledge. The survey highlighted the need for promotion of other healthcare professions in schools and beyond. In England, the school system comprises: primary education (ages five to 11) and secondary education (ages 11 to 16). Further Education (FE) is for those aged over 16 and includes FE colleges, sixth form colleges and work-based learning. Higher education is at a higher level than FE which leads to a qualification/credit awarded by a degree-awarding body. If an adult does not have traditional qualifications they can undertake an Access Course to prepare them for university study, for example if they are career changers or returning to education.

Individuals rely on various sources, such as careers advisors, television programmes, previous healthcare roles or work-shadowing opportunities, that influence their decision to pursue a career in one of the AHPs. To date, previous research exploring which sources of influence affect AHP career choice has been based in Australia [13], Canada [14] or the United States [15]. In the study by Byrne [13] exploring the influence of exposure to OT in career choice for current OT students through a questionnaire, personal or professional exposure to the profession was key. In a questionnaire exploring career choice to current dietetics students [14], most chose this profession at college or university. The media was seen as a key initial source of information about dietetics, with family members the most important person in influencing career choice. Exploring career choice among 312 qualified art therapists [15], talking to an art therapist or a friend had been the reasons for pursuing a career in art therapy. In two studies by Craik [16, 17], exploring career choice of first year OT students at a university in England, work in a healthcare setting and an occupational therapist/student family member/friend were the two most popular methods for first hearing about the profession, with an employers’ career fair the least popular. Making use of a university/college prospectus was key to pursue initial interest, with websites ranking the lowest.

These studies focused on an individual profession and there are also a noticeable proportion of other studies in this topic area which took place pre-2000. There are few UK-based studies on this topic, with the exception of Craik’s work [16, 17]. Although exploring different sources which students or qualified AHPs had utilised to make their career choice, the focus of the studies tended to be when the participants first heard about the profession. Two studies have been conducted to explore strategies for supporting recruitment across various AHPs [18, 19]. However, these studies did not encompass all AHP professions and also included nursing and midwifery and were for particular demographics: mature students [18] and male students [19].

To our knowledge, there has not been a research study exploring the sources of influence which impact career choice across all the AHPs, according to the definition used in England, through a questionnaire. The aim of this study therefore was to understand the influence of sources which drive the career choices and the stage in which AHP students in England are choosing their profession to identify recommendations for stimulating demand for choosing an AHP career.

## Methods

The questionnaire was disseminated to all pre-registration AHP students (more details below). This paper reports on the findings across all the AHPs. Previous publications used a subset of the dataset to focus on two AHPs in need of improving recruitment in England: ODPs [20] and podiatrists [21].

### Sample

Eligible participants were current pre-registration undergraduate and postgraduate students, and apprentices on pre-registration AHP courses in England at the time of the questionnaire. Individuals yet to start their course or who had already graduated were excluded. In England, art therapy, music therapy and dramatherapy are only available as postgraduate courses.

### Ethical approval

Ethical approval was obtained from the University of Winchester’s Research and Knowledge Exchange Ethics Committee (Reference: HWB_REC_21_03). Participant information explaining the study was included at the start of the questionnaire. Informed consent from participants was taken through the ticking of a consent box before the questionnaire. Participation in the questionnaire was kept confidential and anonymous. The study was conducted in accordance with the Declaration of Helsinki.

### Data collection

The online questionnaire, hosted by JISC (Bristol, UK), was available between February and March 2021. Convenience sampling comprised the questionnaire being disseminated, via an access link, to universities and practice placement environments by HEE Education leads for each AHP. Additionally, there was promotion of the questionnaire through social media, the HEE website and HEE newsletters.

The questionnaire content was informed by a scoping review [22] and focus groups undertaken with AHP students. The questionnaire was piloted by 50 physiotherapy students at the University of Winchester, members of an AHP leadership programme and the HEE Education leads. To establish face validity, those piloting the questionnaire gave feedback on the readability and relevance of the questions.

The questionnaire (available as supplementary material) comprised both open and closed questions. There were five sections: demographics, motivations, sources of influence, barriers affecting AHP career choice and four open questions. The focus of this paper is sources of influence as this is most applicable to addressing recruitment to the AHPs to support national policy development and sustainable workforce pipelines. There were four categories of sources of influence: personal, educational, professional and media. For this part of the questionnaire, 5 point Likert-scale questions were used: strongly disagree (1), disagree (2), neutral (3), agree (4), strongly agree (5). Additionally, participants could choose ‘not applicable’, which they were asked to select if they had no contact with the source; our focus was to understand the influence of sources which individuals *had* experienced. There was also a free text box available after each section for any further comment. Completion of the questionnaire took approximately fifteen minutes.

### Analysis

Statistical analysis was primarily descriptive. Differences between sources of influence and gender, ethnicity and age (< 21 or ≥ 21 years) were tested using Pearson’s Chi-square test. To do this, the 5-point Likert scale was dichotomised to 1 = strongly disagreed, disagreed and neutral and 2 = strongly agreed and agreed [23]. Participants who selected ‘not applicable’ were set as missing data. Statistical significance was set at *p* < 0.05 throughout. SPSS (v.29) was used for the statistical analysis. Due to the low number of participants in art therapy, music therapy, and dramatherapy, these were grouped into a single category: (AMDT).

## Results

A total of 1,326 participants completed the questionnaire; however, eight were excluded as they were not current students. This resulted in a final sample of 1,318 participants.

**Table 1:** Demographics and profession

Demographic characteristic	Classification	Number (%)
Participants		**1318**
Gender^a^	Female Male	1055 (80%) 254 (19.3%)
Ethnicity^b^	White Asian background	1080 (81.9%) 107 (8.1%)
	Mixed or Multiple ethnic	
	Background	53 (4%)
	Black, African or Caribbean	
	Background	47 (3.6%)
	Other background	30 (2.3%)
Age	Under 21 21–30 31–40 41–50 51 and over	326 (24.7%) 560 (42.5%) 247 (18.7%) 142 (10.8%) 43 (3.3%)
Type of study^bcd^	Undergraduate Postgraduate Apprenticeship	1102 (83.6%) 175 (13.3%) 36 (2.7%)
Profession	AMDT Podiatry Dietetics OT ODP Orthoptics Osteopathy Paramedic Science Physiotherapy Prosthetics/Orthotics Radiography (Diagnostic) Radiography (Therapeutic) SLT	41 (3.1%) 115 (8.7%) 106 (8%) 131 (9.9%) 153 (11.6%) 26 (2%) 56 (4.2%) 254 (19.3%) 134 (10.2%) 26 (2%) 93 (7.1%) 62 (4.7%) 121 (9.2%)

a Non-binary, other and not disclosed all cell count under 5

b One participant did not disclose ethnicity or year of study

c One participant did not disclose year of study

d Other – cell count under 5

Table 1 shows the demographics and professions of the questionnaire participants. In terms of gender, 80% of the sample (*n* = 1,055) were women, 19.3% (*n* = 254) were men and 0.4% (*n* = 5) were non-binary. The majority of participants were of white ethnicity (*n* = 1080, 81.9%). The highest number of participants were aged between 21 and 30 years (*n* = 560, 42.5%), which would include students in their final year and mature students in any year. Of the participants, 83.6% (*n* = 1102) were on undergraduate courses, with 13.3% (*n* = 175) on postgraduate courses, 2.7% (*n* = 36) on apprenticeship routes and 0.2% (*n* = 3) chose ‘other’. Within our sample, the highest number of participants were those from paramedic science (19.3%, *n* = 254) and the fewest participants were from orthoptics and prosthetics/orthotics (2%, *n* = 26).

Table 2 highlights at what stage participants were choosing their profession. The two most popular stages were after a first career (32.5%, *n* = 427) and during college/sixth form (26.6%, *n* = 350). During primary education was chosen by the fewest participants (1.4%, *n* = 18), with only paramedic science having more than five participants choose this option. There was variation in the stages between professions. For example, at the secondary education stage, 3.8% (*n* = 5) of OT participants chose an OT career compared to 20% (*n* = 27) of physiotherapy participants. Choosing an AHP career after a first career was applicable to over 20% of participants for each profession (range: 21.7% for dietetics − 45.8% for OT). Choosing the profession during a first degree was highest for SLT participants with this being applicable to 23.1% (*n* = 28). Six professions had fewer than five participants choose this option. University clearing in England is how universities and colleges fill spaces available on courses. Applicants can use clearing if they do not have a current offer of a course place or have not met the requirements for that place. In the total sample, 3.7% (*n* = 48) of participants chose their profession at the university clearing stage. Most cell counts for clearing were under 5 for the professions, with the exception of podiatry (12.2%, *n* = 14), therapeutic radiography (14.5%, *n* = 9) and diagnostic radiography (6.5%, *n* = 6). Other stages for choosing the profession included through an Access Course, working as a healthcare assistant (HCA) or after more than one career. For 30.1% (*n* = 46) of ODP participants, they had chosen this career at an ‘other’ stage with 15 of these participants referring to exposure to working in theatre, as a HCA, theatre support worker or clinical support worker as influencing this choice. In comparison, 6.6% (*n* = 7) of dietetics participants had chosen their career at an ‘other’ stage.

**Table 2:** The stage in which the participant chose their profession

Stage of choosing profession	AMDT	Podiatry	Dietetics	OT	ODP	Orthoptics	Osteopathy	Paramedic Science	Physiotherapy	Prosthetics/ Orthotics	Radiography (Diagnostic)	Radiography (Therapeutic)	SLT	Total (%)
Primary education (up to age 11)	-	-	-	-	-	-	-	8 (3.1%)	-	-	-	-	-	18 (1.4%)
Secondary education (11–16)	-	-	27 (25.5%)	5 (3.8%)	-	-	-	56 (22%)	27 (20%)	6 (23.1%)	6 (6.5%)	-	14 (11.6%)	156 (11.9%)
College/sixth form (16–18)	5 (12.2%)	25 (21.7%)	29 (27.4%)	23 (17.6%)	24 (15.7%)	14 (53.8%)	21 (37.5%)	69 (27.2%)	44 (32.8%)	12 (46.2%)	33 (35.5%)	18 (29%)	33 (27.3%)	350 (26.6%)
During university clearing	-	14 (12.2%)	-	-	-	-	-	-	-	-	6 (6.5%)	9 (14.5%)	-	48 (3.7%)
During first degree	9 (22%)	5 (4.3%)	19 (17.9%)	9 (6.9%)	5 (3.3%)	-	-	-	14 (10.4%)	-	-	-	28 (23.1%)	105 (8%)
After first career	18 (43.9%)	45 (39.1%)	23 (21.7%)	60 (45.8%)	69 (45.1%)	-	16 (28.6%)	79 (31.1%)	33 (24.6%)	-	32 (34.4%)	18 (29%)	32 (26.4%)	427 (32.5%)
Other	7 (17.1%)	20 (17.4%)	7 (6.6%)	30 (23%)	46 (30.1%)	-	9 (16.1%)	35 (13.8%)	13 (9.7%)	-	16 (17.2%)	9 (14.5%)	13 (10.7%)	211 (16%)
Total in profession	41	115	106	131	153	26	56	254	134	26	93	62	121	1315*

-cell count under 5, * Three participants did not disclose stage

**Table 3:** Number (and %) participants who agreed/strongly agreed different sources had affected their career path

Source of influence	Overall no. who answered question^a^	Agree/strongly agree (%)	Number (%) of participants < 21	Number (%) of participants *≥* 21	Person Chi-square^b^	Number (%) of ethnic minority participants	Number (%) of white participants	Person Chi-square^b^
Personal sources								
Role model	1085	798 (73.5%)	185 (72.8%)	613 (73.8%)	0.768	133 (68.2%)	665 (74.7%)	0.062
Family member/relative	928	434 (46.8%)	122 (53%)	312 (44.7%)	**0.028**	86 (50%)	347 (46%)	0.338
Friend	917	365 (39.8%)	73 (34.1%)	292 (41.5%)	0.052	68 (40.2%)	296 (39.6%)	0.883
Someone who works closely with profession	937	483 (51.5%)	106 (48.2%)	377 (52.6%)	0.253	97 (56.4%)	386 (50.5%)	0.159
Own/relative experience as patient	946	540 (57.1%)	134 (58%)	406 (56.8%)	0.744	100 (59.2%)	440 (56.6%)	0.545
Own research	1250	1098 (87.8%)	281 (89.8%)	817 (87.2%)	0.226	194 (86.2%)	903 (88.2%)	0.415
Education sources								
Teacher	815	172 (21.1%)	64 (28.3%)	108 (18.3%)	**0.002**	39 (25.7%)	133 (20.1%)	0.127
Profession visiting school/college	748	143 (19.1%)	42 (21.5%)	101 (18.3%)	0.317	36 (25.2%)	107 (17.7%)	**0.041**
Future careers programme	742	121 (16.3%)	53 (25.4%)	68 (12.8%)	**< 0.001**	38 (25.9%)	83 (13.9%)	**< 0.001**
Careers advisor	770	126 (16.4%)	53 (24.9%)	73 (13.1%)	**< 0.001**	41 (25.9%)	85 (13.9%)	**< 0.001**
First university degree	741	281 (37.9%)	28 (21.9%)	253 (41.3%)	**< 0.001**	59 (41.8%)	222 (37.0%)	0.286
Professional sources								
Voluntary work in healthcare	865	434 (50.2%)	133 (59.1%)	301 (47%)	**0.002**	86 (52.1%)	348 (49.7%)	0.578
Work shadowing with profession	964	601 (62.3%)	183 (70.1%)	418 (59.5%)	**0.002**	114 (61.6%)	486 (62.5%)	0.831
Previous job in healthcare	765	375 (49%)	36 (27.3%)	354 (55.9%)	**< 0.001**	79 (58.1%)	296 (47.1%)	**0.020**
Media sources								
Seeing people like me doing profession represented in media	969	368 (38%)	105 (41.8%)	263 (36.6%)	0.144	58 (33.7%)	310 (38.9%)	0.205
Social media	1017	382 (37.6%)	111 (40.8%)	271 (36.4%)	0.196	71 (38.8%)	311 (37.3%)	0.703
The WOW show	646	21 (3.3%)	9 (5.6%)	12 (2.5%)	0.056	6 (4.9%)	15 (2.9%)	0.258
Television programmes	858	221 (25.8%)	80 (35.4%)	141 (22.3%)	**< 0.001**	33 (22.3%)	188 (26.5%)	0.290
Print adverts	788	62 (7.9%)	18 (9.1%)	44 (7.5%)	0.460	21 (14.2%)	41 (6.4%)	**0.002**
TV adverts	808	93 (11.5%)	30 (14.2%)	63 (10.6%)	0.161	18 (12.4%)	75 (11.3%)	0.707
National bodies	927	302 (32.6%)	78 (32.8%)	224 (32.5%)	0.941	46 (28.6%)	256 (33.4%)	0.233
Information from university	1151	825 (71.7%)	235 (77.3%)	590 (69.7%)	**0.011**	155 (74.2%)	670 (71.1%)	0.378
University open day	1099	794 (72.2%)	239 (79.1%)	555 (69.6%)	**0.002**	135 (69.9%)	659 (72.7%)	0.432
Careers Fair	829	249 (30%)	100 (41.5%)	149 (25.3%)	**< 0.001**	62 (38.8%)	187 (28%)	**0.007**
Profession national day	766	164 (21.4%)	46 (23%)	118 (20.8%)	0.524	38 (27.1%)	126 (20.1%)	0.067

a Total number of participants who had answered the question, excluding participants who selected ‘not applicable’

b Chi-square tested the difference between participants’ age/ethnicity who agreed/strongly agreed compared to those who strongly disagreed/disagreed/neutral to the statement

As shown in Table 3, *Conducting your own research* was the most influential with 1098 participants agreeing/strongly agreeing with this source, which represented 87.8% of participants who answered the question. This source is likely to comprise a number of resources such as *social media* and *information from universities* and is not a single external source of influence. Other highly influential sources of influence were *a role model in the profession* (73.5%, *n* = 798), *a university open day* (72.2%, *n* = 794) and *information from universities* (71.7%, *n* = 825). Sources with less influence included: *the WOW show* (3.3%, *n* = 21), *print adverts* (7.9%, *n* = 62), *television adverts* (11.5%, *n* = 93), *careers advisor* (16.4%, *n* = 126) and *someone in the profession visiting school/college* (19.1%, *n* = 143).The WOW show, an online channel introducing young people to different careers, was launched in 2019 and therefore it is likely that a proportion of questionnaire participants had applied and/or begun their course before 2019.

When comparing participants by gender, the only statistically significant difference was in relation to a *careers fair* with this source being more important for female participants. There was one significant difference in the use of ‘personal sources’ when comparing participants aged < 21 and ≥ 21 years, and between different ethnicities (both *p* > 0.05), a *family member/relative* was more important for participants aged < 21 than ≥ 21 (*p* = 0.028). When considering education sources, significant differences were observed between participants aged < 21 and those ≥ 21 years with regards to the *futures careers programme* (25.4% vs. 12.8%, respectively *p* ≤ 0.001). Similar findings were observed when comparing ethnic minority participants with white participants (25.9% vs. 13.9%, respectively *p* ≤ 0.001). The influence of a *careers advisor* was important for 24.9% of those aged < 21 in comparison to 13.1% of participants aged ≥ 21 years (*p* ≤ 0.001), although it cannot be determined when this interaction took place: whether during school/college or another setting. In addition, a *careers advisor* was strongly influential for 25.9% of ethnic minority participants compared to 13.9% of white participants (*p* ≤ 0.001). The influence of a *careers advisor* was highest for the paramedic sciences (n = 27) and dietetics (n = 18) in terms of participants. For other professions such as podiatry (n = 9) and for therapeutic radiography (n = 6) the numbers were much lower. Across the professions, 41.6% (n = 548) did not receive information from a *careers advisor*, with 16.4% (n = 126) strongly agreeing/agreeing with a *careers advisor* as a source of influence. For 41.3% (n = 253) of those aged ≥ 21 they agreed/strongly agreed with the influence of *completing a first university degree* (*p* ≤ 0.001).

Professional sources of influence were statistically significant between the two age groups. *Voluntary work in healthcare* had influenced 59.1% of participants aged < 21 compared to 47% of those ≥ 21 (*p* = 0.002). *Work shadowing with the profession* was influential for 70.1% of those aged < 21, in comparison to 59.5% of those aged ≥ 21 (*p* = 0.002). This represented 183 of the total 326 participants (56.1%) in the < 21 age group and 418 out of the total 992 participants (42.5%) in the ≥ 21 age group. *A previous job in healthcare* was more important for participants aged ≥ 21 (55.9%) compared to the participants aged < 21 (27.3%) (*p* ≤ 0.001).

For media sources, *print adverts* were statistically significantly more important for ethnic minority participants (14.2%) than white participants (6.4%) (*p* = 0.002). In terms of age, *television programmes* and *career fairs* were statistically significant for those aged < 21 (35.4%; 41.5%) compared to participants aged ≥ 21 (22.3%; 25.3%) (*p* ≤ 0.001). Finally, we found that 79.1% of participants aged < 21 had been influenced by *a university open day* compared to 69.6% of participants aged ≥ 21 (*p* =0.002).

## Discussion

This study explored the findings of a national questionnaire among AHP students in England examining the influence of sources and stages in which they are making their career choice. *Conducting their own research*, *impact of a role model* and *university open days* and *information from university* were all the most influential sources, however, sources such as a *careers advisor*, *a futures careers programme* or *print adverts* and *television adverts* were the least influential. Differences in the influences of the sources were found between age and ethnicity. Our findings suggest the opportunity to utilise less influential sources more effectively (for example, *careers advisors*) but also to continue to focus on mechanisms that are working effectively (*role models* and *information from universities*). Our analysis did confirm, as would be expected, that most AHP students are making their career choice during sixth form/college or after a first career. However, there were low and varied levels of exposure to AHPs at primary and secondary school. Understanding more as to why AHP students choose their careers will help to produce a more targeted approach to recruitment strategies at local and national level. This can help meet the supply needs of the AHPs to meet the healthcare demands of the population going forward.

This study focused on the influence of sources but there is a more general need to increase the visibility of the AHPs among different sources of influence. Without this, it becomes more difficult for individuals to choose an AHP career. Previous studies have found a lack of awareness about the AHPs as a key barrier to choosing their career [18] and specifically for ODP [20, 24] and podiatry [21, 25]. This suggests that effort is needed to enable the different sources of influence to promote these professions. This could be done through utilising three key sources: careers advisors, role models as career ambassadors and universities.

### Careers advisors

Our study supports the findings presented in a recent scoping review [22] of careers advisors as a low source of influence. This was evident despite college/sixth form being the second most popular stage for choosing an AHP career. The Office for Students study [19] identified an opportunity for the promotion of more varied healthcare careers and more effective career guidance. The low numbers in our findings among professions such as podiatry and therapeutic radiography suggest that careers advisors are *not* currently key influencers for these professions. The level of influence of careers advisors in our study was higher for two of the better known professions (dietetics and paramedic sciences) although this was still relatively low in comparison with other sources.

In the study by Lordly and Dube [14], they found that guidance counsellors were not influential for dietetics students but highlighted the importance of determining whether the students never received information from the guidance counsellor, or that the information they *did* receive was not influential. Across all the professions in our sample, 41.6% did not receive information from a careers advisor, with only 16.4% strongly agreeing/agreeing with a careers advisor as a source of influence for choosing their profession. These findings suggest a need to improve the provision and quality of careers advice. Future research into how best to support careers advisors is recommended. Additionally knowing there are different paths to becoming an AHP [26] is important for careers advisors to convey. Our study identified a statistical significance in the level of influence of careers advisors for ethnic minority participants in comparison to white participants. This suggests the potential impact of careers advisors and owing to the lack of ethnic diversity in a number of the AHPs [27], ensuring effective utilisation of careers advisors could be beneficial in addressing this.

### Role models as career ambassadors

Similar to our findings, in the literature, qualified professionals acting as role models were identified as an important source of influence. This was recognised as a dual role by Craik and Zaccaria [16] suggesting that current OTs should promote the profession to those who are unaware *and* to those who already have an interest in the profession. Accordingly, it is important that current AHPs are made aware of their responsibility as a role model to inspire others [17]. One of the most common sources for qualified art therapists becoming interested in choosing this career was through talking with an art therapist [15].

There is published guidance on how to promote AHP careers and engage in outreach work including within schools and colleges [26]. Since 2018, there has been an NHS Ambassadors scheme comprising NHS staff promoting their profession to school pupils. In the NHS Ambassadors Evaluation report whilst the School Leads reported that NHS Ambassador visits had a positive impact on the students, there was concern from the NHS Ambassadors that schools were unaware of the variety of different healthcare roles in the NHS with some ambassadors not being approached [28]. Accordingly, it would be beneficial to equip AHP NHS Ambassadors to promote other AHPs beyond their own. For example, a physiotherapist explaining how they are part of the AHPs and similar professions in the AHP family include podiatry, OT and prosthetics/orthotics. This would require those staff to have effective supporting resources to feel capable and confident to have these conversations.

Our study found that the highest proportion of students were choosing their AHP career after a first career (32.5%, *n* = 427). Whilst this finding highlights the need to focus resources on students learning about the AHP professions in school/college, there should still be resources targeted at the ‘career changers’ group. The ‘Strategic Interventions in Health Education Disciplines’ (SIHED) included the creation of online Continuing Professional Development (CPD) training courses for AHPs to complete to become volunteers in outreach work with career changers as well as young people [29]. A HEE funded initiative from Macmillan and the Society and College of Radiographers, focused on promoting therapeutic radiography as a career including hosting career events for potential course applicants [30]. This highlights that role models are recognised key sources of influence and similar initiatives should continue to be utilized.

Our findings also showed that a previous job in healthcare was influential for those aged over 21 years; exploring how the AHPs can be promoted effectively in this setting is an opportunity for career ambassadors. Those who had previously worked in healthcare may include individuals who discover the less well-known AHPs and pursue this career through an apprenticeship; this is gaining importance as reflected in the stretching apprenticeship targets in the NHS Long Term Workforce Plan [7]. Work shadowing was influential in our sample but access is a barrier: for those aged under 21 years only 183 of 326 participants (56.1%) had exposure to this source. Indeed in the 2024 Youth Voice Census Report, a key finding was young people’s anxiety about their lack of work experience [31]. For ODPs, accessing observational clinical visits and work experience were identified as key challenges [20, 32]. One way of addressing this is through virtual work experience. The Aspiring Allies campaign [26] involved virtual work experience for college students to learn about the AHPs through completing modules in practical skills. Looking at health professions beyond AHPs, those interested in a career in medicine aged 16 and over can use ‘Observe GP’ as virtual work experience which is a video platform sharing insights into a day in the life of a GP (General Practitioner) and the primary care team [33].

### Universities

A *university open day* (72.2%, *n* = 794) and *information from universities* (71.7%, *n* = 825) were two of the most influential sources of influence for choosing an AHP career in our sample. Beyond informing prospective students at school/college and career changers about the AHP courses, we suggest two further ways in which universities could be utilised: to introduce career awareness to the AHPs as part of support of local schools and colleges aligned to the Gatsby benchmarks [34] and to current university students who may select an AHP for MSc Pre-registration. Gatsby benchmarks are a framework which secondary schools and colleges in England can use to evidence relating learning in the classroom to careers. They are designed to embed and enhance careers work in the national curriculum in schools. There is significant opportunity for universities collaborating with the professions and local NHS and wider healthcare services, to engage in career awareness through the school curriculum so students are exposed to the AHPs at an earlier age. Our study found only 174 (13.2%) of participants chose their career during primary or secondary education.

The most popular stage of choosing dietetics in the study by Lordly and Dube [14] was at college (the UK equivalent to university). For our study, using a different definition, we found choosing their AHP career during their first degree was a less popular stage, suggesting an opportunity to utilise sources of influence to current university students. Current students on AHP courses at the university could be utilised to promote their chosen profession as well as promotion of AHP postgraduate courses. Universities have a vested interest in leaver destinations in terms of regulation from the Office for Students and therefore providing effective career advice both before students choose their university course and during will be beneficial.

Following our study findings, we recommend that:

1. The Primary School curriculum is supported with related careers exposure to wider professions or wider topics e.g. care and rehabilitation.
2. Careers advisors, or those providing career advice, at secondary school/college or within career changers advice services are equipped with resources to have greater awareness of the AHPs, skills to map an individual’s interest to a wider range of healthcare professions and resources with effective signposting to learn more/speak to someone in the profession.
3. Further work is needed to coproduce effective strategies with underrepresented communities to support meaningful and impactful careers information, support and potential mentorship. In addition, future research should explore the impact of careers advisors and related interventions with learners with protected characteristics to support career choices and build professional diversity.
4. Work experience/volunteering/T level (technical qualifications recognised in England for students aged 16–19) or work placements should be easily accessible within local communities in person [35].
5. AHP Career Ambassadors are well informed and supported to feel confident to provide information across all AHPs with effective high quality assets and information that also support schools’ careers activity.
6. Universities and appropriate organisations are encouraged to promote the diverse range of AHP careers to young people during school years supported by national resources that can form part of the curriculum and meet the national policy that schools need to evidence e.g. Gatsby Benchmarks [34].
7. University career services and those responsible for placements/volunteering opportunities are supported by effective resources and information to promote postgraduate AHP courses and AHP careers to current students on non-health programmes.
8. Outreach work to target mature students or career changers in settings that align with the AHPs: for example the military, sports or education sectors.

### Strengths and limitations

A strength of this study is that it adds to the knowledge base around how AHP students are choosing their profession. Our study is among the first to investigate the sources of influence on choosing an AHP career across all the professions, enabling us to analyse the findings both collectively and by individual profession. Additionally, we had representation from all the AHPs and a large sample size.

However, our study had limitations. The questionnaire was piloted among both AHP students and professional bodies but it had not been validated. Our questionnaire was disseminated online and comprised a self-selecting sample which has limitations [36] and therefore cannot be seen to represent the views of all AHP students in England. Further, there was variation in the number of participants representing each profession, with limited participants from some AHPs. This should be acknowledged in relation to findings for ‘Primary education’ and ‘During university clearing’. Participants were able to provide a ‘not applicable’ answer and were instructed to choose this option if they had not been influenced by a source. It is likely that some participants instead chose ‘strongly disagree’ which may have impacted our findings. However, this was mitigated through our focus on ‘strongly agree/agree’. Finally, data collection took place in 2021. Since this study was undertaken, wider development of support roles, T levels, apprenticeships and better community immersion through anchor organisations offer a step change in attraction to the NHS. Accordingly, our respondent population may be different now and certain sources may be more influential. Use of social media and its different forms has grown since data collection. However individuals are likely to use alternative sources to find out about a career and therefore our results in relation to social media are unlikely to noticeably change.

## Conclusions

Attracting people to poorly visible professions requires significant attention. Many parties share the responsibility of attracting the next generation of AHPs. This study shows the multiplicity of sources but also missed opportunities to introduce people to wider health workforce roles. Limited resources require strategic partnerships to maximise impact and all parties maximising their contribution. For example, current AHPs as role models have the opportunity to be influential in a number of different forms through having an openness to a conversation about their career. They can: recognise that every personal conversation impacts someone’s desire to follow the career or suggest their child, relative or friend follow the career; attend school/community career events and utilise high quality careers information developed and made available through the NHS and professional bodies; and offer work experience availability and T level placements. This then leads individuals to access university careers information. This route needs to offer the next level of attraction and focus on exposing people to varied other professions and information to support alternative related choices if unsuccessful, as well as supporting BSc students to consider MSc healthcare routes at their own and other universities.

Further work is needed on schools’ awareness and support to careers advisors, teachers and parents as this was a significant missing link. The developing careers work of professional bodies anchored in the Gatsby Benchmarks to aid utility for schools is key to linking understanding of the professions to aspects of the national curriculum. Further work on targeted approaches to career changers e.g. the military, police, elite sports people are also showing early signs of help with addressing future AHP workforce shortages in England. A joined-up approach utilising these sources of influence will be needed to meet this demand.

## Supplementary Information

Below is the link to the electronic supplementary material.

Supplementary Material 1

## Data Availability

The datasets used and/or analysed during the current study are available from the corresponding author on reasonable request.

## Abbreviations

AHP: Allied Health Professionals
AMDT: Art therapy, music therapy, and dramatherapy
CPD: Continuing Professional Development
FE: Further Education
GP: General Practitioner
HCA: Healthcare Assistant
HCPC: Health and Care Professions Council
HEE: Health Education England
NHS: National Health Service
ODP: Operating department practitioners
OT: Occupational therapy
SIHED: Strategic Interventions in Health Education Disciplines
SLT: Speech and language therapists
UK: United Kingdom
