# Supplementary material for: What drives career choice in allied health professions in England? Insights from a national questionnaire
Source: BMC Health Serv Res. 2025 Dec 25;26:373. doi: 10.1186/s12913-025-13591-y (PMC13001238; doi:10.1186/s12913-025-13591-y)
Supplement: Supplementary file 1 — Supplementary Material 1 [file 12913_2025_13591_MOESM1_ESM.pdf]

Motivations for choosing an allied health profession career

Page 1: Participant Information

Please read this information carefully before deciding to take part in this questionnaire.

You are invited to take part in a research study. Before you decide to take part it is important for you to understand why the research is being done and what it will involve. Please take time to read the following information carefully.

What is the research about?

This is a Health Education England and University of Winchester project exploring motivations for and the decision making process in choosing an Allied Health Profession (AHP) career.

Health Education England provide national leadership and coordination for the education and training within the health and public health workforce within England. AHPs represent the third largest workforce in health and care in England and comprise 14 professions: art therapists, dietitians, drama therapists, music therapists, occupational therapists, operating department practitioners (ODPs), orthoptists, osteopaths, paramedics, physiotherapists, chiropodists and podiatrists, prosthetists and orthotists, diagnostic and therapeutic radiographers, and speech and language therapists.

Choosing a career in allied healthcare is a life changing decision however it is not clear what factors are the key influencers on decision making. We know that there is considerable interest in healthcare careers and there is competition for places. As students on an allied healthcare programme, you are already embarking on a career in the allied health professions and you will be aware of the challenges in preparing students for employment within this sector. Whilst much is known about the academic skills and critical clinical decision making skills that students require, the factors that drive students to consider a career as an AHP are the subject of considerable debate.

This questionnaire consists of demographic questions and questions exploring the impact of different motivations on choosing your AHP career, sources of influence on your decision making and barriers you had to overcome to choose this career.

What will happen to me if I take part?

This questionnaire aims to gain baseline information about your views, the factors influencing your views and your decision making process. The questionnaire will take approximately 15 minutes to complete and mainly comprises questions asking you to rank your perceptions on a scale.

Are there any benefits/risks involved?

There may be no benefit to the individual, but the main aim of this project is to explore the factors that drive you to consider a career as an AHP. The NHS Long Term Plan requires a significant increase in AHP workforce and therefore investigating this area will inform future AHP recruitment strategy in England. This is a national questionnaire distributed to all students enrolled in AHP courses in England. Therefore, your involvement allows you to represent your AHP and raise its profile. It will help us to develop better information for healthcare career promotion and advice.

There are no risks from taking part. Information and identity will remain confidential and only be known to the researchers.

Why have I been chosen?

You have been chosen because you are already embarking on a career in the allied health professions through being a student on an AHP course.

Do I have to take part?

You do not have to take part, however for those willing to, it would be gratefully appreciated if you can complete all questions in the questionnaire.

Will my participation be confidential?

This study will comply with GDPR policy. Any information will be stored as hardcopy and electronically. Your answers will be treated as confidential and will be anonymised for the purposes of analysis and write-up.

What happens if I change my mind?

Any participant has the right to withdraw at any time without their legal rights being affected. The timeframe for withdrawal from the questionnaire would be 3 weeks after submission of the questionnaire. Intention to withdraw from the study should be made by email to the lead researchers.

What happens if something goes wrong?

If you have a concern, or complaint, about this study you should contact the lead researchers of Dr Lucy Wallis, Dr Rachel Locke or Dr Steve Ryall. Contact details are lucy.wallis@winchester.ac.uk, Rachel.Locke@winchester.ac.uk or Steve.Ryall@winchester.ac.uk. If you any concerns regarding your involvement in this research, please discuss these with the researchers in the first instance. If you remain unhappy about the study and wish to make a complaint, please contact Amy.Wright@winchester.ac.uk (Faculty Ethics Lead, Faculty of Health and Wellbeing) at the University of Winchester.

Where can I get more information?

More information can be obtained from the lead researcher, Dr Lucy Wallis, who will answer any questions that you may have after reading the information sheet.

Page 2: Consent

I have read the participant information page and I am happy to participate. I understand that by completing and submitting this questionnaire I am consenting to be part of the research study and for my data to be used as described. ✱ *Required*

- [ ] I agree

Page 3: Background/Demographics

What is your gender?

- Male
- Female
- Non-binary
- Other

Are you studying full-time or part-time?

- Full-time
- Part-time

Which year of study are you in?

- Undergraduate First Year
- Undergraduate Second Year
- Undergraduate Third Year
- Undergraduate Fourth Year
- Undergraduate Fifth Year
- Undergraduate Sixth Year
- Postgraduate First Year
- Postgraduate Second Year

What is your age band?

- Under 21
- 21-25

- 26-30
- 31-35
- 36-40
- 41-45
- 46-50
- Over 51

Which option best describes your ethnic group? (Source: gov.uk)

- English, Welsh, Scottish, Northern Irish or British
- Irish
- Gypsy or Irish Traveller
- Any other White background
- White and Black Caribbean
- White and Black African
- White and Asian
- Any other Mixed or Multiple ethnic background
- Indian
- Pakistani
- Bangladeshi
- Chinese
- Any other Asian background
- African
- Caribbean
- Any other Black, African or Caribbean background
- Arab
- Any other ethnic group
- Other

If you selected Other, please specify:

Are your day-to-day activities limited because of a physical or mental health problem or disability which has lasted, or is expected to last at least 12 months? Include limitations you would experience without medication or treatment and limitations that are due to old age. (Source: Development of disability questions: 2011 Census for England and Wales & Integrated Household Survey)

- ○ Yes, limited substantially
- ○ Yes, limited but not substantially
- ○ No

If you have responded 'Yes' above. Do you have any of the following impairments or health conditions?

- ☐ a physical impairment such as difficulty using your arms or mobility difficulties which require you to use a wheelchair or other mobility aid
- ☐ a sensory impairment such as serious vision difficulties or blindness, or deafness
- ☐ a mental health condition, such as depression or schizophrenia that has lasted or is expected to last 12 months or more
- ☐ a learning difficulty or disability such as down's syndrome or dyslexia or a cognitive impairment such as autistic spectrum disorder
- ☐ diagnosed as having HIV, Cancer or Multiple Sclerosis
- ☐ Other long-term illness or health condition that has lasted or is expected to last 12 months or more
- ☐ N/A

My chosen professional area is:

- ○ Art Therapy
- ○ Chiropody/Podiatry
- ○ Dietetics
- ○ Drama Therapy
- ○ Music Therapy

- Occupational Therapy
- Operating Department Practice
- Orthoptics
- Osteopathy
- Paramedic Science
- Physiotherapy
- Prosthetics/Orthotics
- Radiography (Diagnostic)
- Radiography (Therapeutic)
- Speech and Language Therapy

Which other health professions did you consider? (Please tick all which apply).

- [ ] Art Therapy
- [ ] Biomedical Science
- [ ] Chiropody/Podiatry
- [ ] Clinical Psychology
- [ ] Dentistry
- [ ] Dietetics
- [ ] Drama Therapy
- [ ] Medicine
- [ ] Music Therapy
- [ ] Nursing
- [ ] None
- [ ] Occupational Therapy
- [ ] Operating Department Practice
- [ ] Orthoptics
- [ ] Osteopathy
- [ ] Other
- [ ] Paramedic Science
- [ ] Pharmacy
- [ ] Physician Associate

- [ ] Physiotherapy
- [ ] Prosthetics/Orthotics
- [ ] Psychology
- [ ] Radiography (Diagnostic)
- [ ] Radiography (Therapeutic)
- [ ] Speech and Language Therapy

If you selected Other, please specify:

At what stage did you make the decision to join this profession?

- ○ Primary education (up to age 11)
- ○ Secondary education (11-16)
- ○ College/Sixth form (16-18)
- ○ During university clearing
- ○ During first degree
- ○ After first career
- ○ Other

If you selected Other, please specify:

When accessing information about your profession which website(s) did you use? (Please tick all which apply).

- [ ] The Health Education England website

- ☐ The NHS Health careers website
- ☐ The website of the professional body for the profession
- ☐ An NHS Trust website
- ☐ I see the difference website
- ☐ The National Careers Service website
- ☐ The UCAS website
- ☐ The Prospects website
- ☐ A university website
- ☐ Facebook
- ☐ Twitter
- ☐ Instagram
- ☐ Youtube
- ☐ Other social media
- ☐ Other

If you selected Other, please specify:

Page 4: Your motivations for choosing your profession

This section explores the extent to which different motivations (personal, professional and the day-to-day content of the job) affected your decision to choose an AHP career.

Below are 10 statements about how your perceptions about your *personal interests and affinities* could influence your AHP career choice. For each statement mark the answer that best describes how you feel. My career choice was influenced by choosing a profession...

Please don't select more than 1 answer(s) per row.

Please select at least 10 answer(s).

	Strongly Disagree	Disagree	Neutral	Agree	Strongly Agree	Not applicable
That reflects my academic interests and abilities	☐	☐	☐	☐	☐	☐
Based on my interest in a particular area of the profession (e.g. art, language or technology)	☐	☐	☐	☐	☐	☐
That is challenging/demanding	☐	☐	☐	☐	☐	☐
That is intellectually stimulating	☐	☐	☐	☐	☐	☐
With high-profile responsibilities	☐	☐	☐	☐	☐	☐
That suits my personal qualities and values	☐	☐	☐	☐	☐	☐
That has a good public image and prestige	☐	☐	☐	☐	☐	☐
Which is respected in my culture	☐	☐	☐	☐	☐	☐

Which is suited to my religious background	☐	☐	☐	☐	☐	☐
Which has supportive attitudes to an individual’s culture and background	☐	☐	☐	☐	☐	☐

Any comments you wish to add relating to your answers:

Below are 15 statements about how your perceptions about the *professional aspect* of your profession could influence your AHP career choice. For each statement mark the answer that best describes how you feel. My career choice was influenced by…

Please don't select more than 1 answer(s) per row.

Please select at least 15 answer(s).

	Strongly Disagree	Disagree	Neutral	Agree	Strongly Agree	Not applicable
Choosing a vocational degree and a profession	☐	☐	☐	☐	☐	☐
Choosing a course that offers student bursaries and finance	☐	☐	☐	☐	☐	☐
Regular working hours of the profession allowing for a good work/life balance	☐	☐	☐	☐	☐	☐
The salary	☐	☐	☐	☐	☐	☐
The potential for job security	☐	☐	☐	☐	☐	☐
The opportunity to be entrepreneurial	☐	☐	☐	☐	☐	☐
Good job availability and employment opportunities	☐	☐	☐	☐	☐	☐

The opportunity to work in the private sector	☐	☐	☐	☐	☐	☐
The opportunity to work in the public sector	☐	☐	☐	☐	☐	☐
The opportunity to work in the NHS	☐	☐	☐	☐	☐	☐
The opportunity to work in healthcare	☐	☐	☐	☐	☐	☐
The ability to move anywhere or to work overseas	☐	☐	☐	☐	☐	☐
The good opportunities for my career advancement	☐	☐	☐	☐	☐	☐
The further educational/research/teaching opportunities	☐	☐	☐	☐	☐	☐
The opportunity to practise in different work environments	☐	☐	☐	☐	☐	☐

Any comments you wish to add relating to your answers:

Below are 10 statements about how your perceptions about the *day-to-day content of the job* could influence your AHP career choice. For each statement mark the answer that best describes how you feel. My career choice was influenced by choosing a profession...

Please don't select more than 1 answer(s) per row.

Please select at least 10 answer(s).

	Strongly Disagree	Disagree	Neutral	Agree	Strongly Agree	Not applicable

Where I can help others and make a contribution to society	☐	☐	☐	☐	☐	☐
Where I can use my skills to improve the quality of life for a patient/service user	☐	☐	☐	☐	☐	☐
Where I can engage in/build human relationships	☐	☐	☐	☐	☐	☐
Where I can work with a range of patients/service users	☐	☐	☐	☐	☐	☐
Where I can help a specific group of people	☐	☐	☐	☐	☐	☐
That is fulfilling and satisfying	☐	☐	☐	☐	☐	☐
That is exciting and involves working under pressure	☐	☐	☐	☐	☐	☐
Which is a non-sedentary profession	☐	☐	☐	☐	☐	☐
Where I can work in a team	☐	☐	☐	☐	☐	☐
That offers variety and is more than just routine	☐	☐	☐	☐	☐	☐

Any comments you wish to add relating to your answers:

Are there any other motivations?

Page 5: Sources of Influence

This section explores the extent to which different sources of influence (personal, educational, media and marketing) affected your decision to choose an AHP career.

Below are 14 *personal and educational* sources of influence impacting on AHP career choice. For each statement mark the answer that best describes how you feel. (Please select N/A if the opportunity did not arise - for example if you did not hear about the profession from a teacher). My career choice was influenced by hearing about the profession through…

Please don't select more than 1 answer(s) per row.

Please select at least 14 answer(s).

	Strongly Disagree	Disagree	Neutral	Agree	Strongly Agree	Not applicable
My previous job in healthcare						
Someone in the profession I saw/met who was a really good role model for me						
A family member or a relative						
A friend						
Someone who works closely with the profession						
My own experience of being a patient/a relative receiving care from the profession						
My own research						
A teacher						

An individual in the profession visiting the school/college	☐	☐	☐	☐	☐	☐
A future careers programme run by my school/college	☐	☐	☐	☐	☐	☐
A careers advice person	☐	☐	☐	☐	☐	☐
Voluntary work in healthcare settings	☐	☐	☐	☐	☐	☐
Work shadowing experiences with the profession	☐	☐	☐	☐	☐	☐
My first university degree	☐	☐	☐	☐	☐	☐

Any comments you wish to add relating to your answers:

Below are 11 *media and marketing* sources of influence impacting on AHP career choice. For each statement mark the answer that best describes how you feel. (Please select N/A if the opportunity did not arise - for example if you did not hear about the profession from a television programme). My career choice was influenced by hearing about the profession through…

Please don't select more than 1 answer(s) per row.

Please select at least 11 answer(s).

	Strongly Disagree	Disagree	Neutral	Agree	Strongly Agree	Not applicable

Seeing people like me doing the profession represented in the media	☐	☐	☐	☐	☐	☐
Social media	☐	☐	☐	☐	☐	☐
The WOW show	☐	☐	☐	☐	☐	☐
Television programmes including sports matches or the news	☐	☐	☐	☐	☐	☐
Print adverts	☐	☐	☐	☐	☐	☐
Television adverts	☐	☐	☐	☐	☐	☐
National bodies	☐	☐	☐	☐	☐	☐
Information I got from universities	☐	☐	☐	☐	☐	☐
Attending a university open day	☐	☐	☐	☐	☐	☐
A careers fair	☐	☐	☐	☐	☐	☐
National days (e.g. National ODP day) at a hospital, university or school/college	☐	☐	☐	☐	☐	☐

Any comments you wish to add relating to your answers:

Are there any other sources of influence?

What do you feel is the public understanding of your profession (positive or negative aspects)?

Page 6: Barriers to entry

This section explores the extent to which potential barriers to entry (personal, professional and understanding of the role) affected your decision to choose an AHP career.

Below are 8 statements about potential *personal* barriers to entering an AHP career. For each statement mark the answer that best describes your experience with the barrier. A potential barrier in my career decision making was presented by...

Please don't select more than 1 answer(s) per row.

Please select at least 8 answer(s).

	Strongly Disagree	Disagree	Neutral	Agree	Strongly Agree	Not applicable
A lack of prestige associated with the profession	☐	☐	☐	☐	☐	☐
Perceived lack of representation in the profession in terms of gender	☐	☐	☐	☐	☐	☐
Perceived lack of representation in the profession in terms of ethnicity	☐	☐	☐	☐	☐	☐
Perceived image of who does the profession	☐	☐	☐	☐	☐	☐
Perceived difficulty of getting onto the course	☐	☐	☐	☐	☐	☐
Perception of the course as difficult (including too academic)	☐	☐	☐	☐	☐	☐

A concern around accessibility of the course/profession in terms of physical or mental disability	☐	☐	☐	☐	☐	☐
The emotional burden of the role	☐	☐	☐	☐	☐	☐
Outside obligations I have	☐	☐	☐	☐	☐	☐

Any comments you wish to add relating to your answers:

Below are 8 statements about potential *professional* barriers to entering an AHP career. For each statement mark the answer that best describes your experience with the barrier. A potential barrier in my career decision making was presented by...

Please don't select more than 1 answer(s) per row.

Please select at least 8 answer(s).

	Strongly Disagree	Disagree	Neutral	Agree	Strongly Agree	Not applicable
Geographical location of training course	☐	☐	☐	☐	☐	☐
Not being able to access funding for a second degree	☐	☐	☐	☐	☐	☐
Funding the course and availability of financial support	☐	☐	☐	☐	☐	☐

Cost of training whilst undertaking the course (e.g. costs of placements)	☐	☐	☐	☐	☐	☐
High workload and pressure of training	☐	☐	☐	☐	☐	☐
Perceived working conditions within the NHS	☐	☐	☐	☐	☐	☐
Attitudes present in the NHS workplace	☐	☐	☐	☐	☐	☐
Job availability	☐	☐	☐	☐	☐	☐

Any comments you wish to add relating to your answers:

Below are 6 statements about potential *understanding of the role* barriers to entering an AHP career. For each statement mark the answer that best describes your experience with the barrier. A potential barrier in my career decision making was presented by...

Please don't select more than 1 answer(s) per row.

Please select at least 6 answer(s).

	Strongly Disagree	Disagree	Neutral	Agree	Strongly Agree	Not applicable
Poor access I had to work shadowing experience	☐	☐	☐	☐	☐	☐
Limited awareness of the existence of the profession	☐	☐	☐	☐	☐	☐

Challenges in accessing information about the profession	☐	☐	☐	☐	☐	☐
Limited amount of information about the profession available	☐	☐	☐	☐	☐	☐
Lack of understanding about the profession from careers advisors at school/college	☐	☐	☐	☐	☐	☐
Misconceptions around the profession and what the role involves	☐	☐	☐	☐	☐	☐

Any comments you wish to add relating to your answers:

Are there any other potential barriers?

Page 7: Career decision making

From what you know now as a student on your course, is there anything that you wish you had known before you began your training course?

From what you know now as a student on your course, what advice would you give someone interested in this profession?

Do you feel you made the right choice of profession?

Page 8: Final page

Thank you very much for completing this questionnaire. Your time and insight is much appreciated.

If you have any questions about the questionnaire or research project as a whole, please email Dr Lucy Wallis at lucy.wallis@winchester.ac.uk

---
